# Adolescents’ Self-Esteem and Life Satisfaction: Communication with Peers as a Mediator

**DOI:** 10.3390/ijerph19073777

**Published:** 2022-03-22

**Authors:** Małgorzata Szcześniak, Iga Bajkowska, Anna Czaprowska, Aleksandra Sileńska

**Affiliations:** 1 Institute of Psychology, University of Szczecin, 71-017 Szczecin, Poland; iga.bajkowska@wp.pl (I.B.); czapranna1a@gmail.com (A.C.); 2 Indywidualna Praktyka Psychologiczna, 71-653 Szczecin, Poland; silenska.a@gmail.com

**Keywords:** adolescents, self-esteem, life satisfaction, peer communication, mediation

## Abstract

The main goal of this study was to verify whether the relationship between adolescents’ self-esteem and life satisfaction is mediated by peer communication. The rationale behind this choice was the fact that while we know a lot about the association between self-esteem and life satisfaction in adolescents, we know far less about the mechanisms that may regulate this direct relationship. The research was conducted among high school students in one of the Polish provincial capitals (*N* = 429). The Rosenberg Self-Esteem Scale, the Satisfaction with Life Scale, and the Scale of Communication of Adolescents with Peers were used. In line with the hypotheses, self-esteem correlated positively with life satisfaction and openness, and negatively with difficulty in communication with peers. Open peer communication was positively associated with life satisfaction, and difficult peer communication was negatively correlated with life satisfaction. Moreover, the association between self-esteem and life satisfaction was mediated by openness and difficulty in peer communication. The mediatory effect of peer communication suggests that the simple bivariate relationship between adolescents’ evaluation of the self and a subjective assessment of their overall quality of life may be more complex.

## 1. Introduction

Adolescents account for 16% of the World’s population [1] and constitute approximately 10% of Polish inhabitants [2]. Adolescence is considered a unique developmental phase [3], characterized by identity uncertainty [4], self-discovery [3], accelerated growth [5], increased autonomy [6], “multiple interactions between biology and culture” [7] (p. 1903), transition from childhood to adulthood [8], socialization [9], and rapid biological [10], psychological [11], social [12,13], and cognitive changes [13,14].

Extensive literature [15] provides evidence that adolescence, as a period of specific alterations in youths’ lives, represents a time of vulnerability which is of great importance to adolescents’ well-being. Although most adolescents report being satisfied with their lives [16,17]), several researchers observed a significant drop of life satisfaction in this age group [15,18,19,20], and consider such a decrease to be a developmental phenomenon [19].

Different studies [3] suggest that adolescents’ life satisfaction depends on a range of personal and social determinants. For example, Huebner et al. [16] listed self-perception and interpersonal relationships among influential predictors of youths’ life satisfaction. Proctor et al. [21], based on a meta-analysis of studies on life satisfaction in adolescence, mentioned self-esteem and relations with peers as its positive correlates. These outcomes are understandable if we take into account that life satisfaction consists of a global assessment that individuals make when they feel fulfilled or happy with their life as a whole or its distinct domains such as the self or relationships [22]. All this considered, the main goal of this study was to verify whether the relationship between adolescents’ self-esteem and life satisfaction is mediated by peer communication. The rationale behind this choice was the fact that while we know a lot about the association between self-esteem and life satisfaction in adolescents, we know far less about the mechanisms that may regulate this direct relationship. Therefore, the novelty of this study consists in the inclusion of peer communication in the relationship between self-esteem and life satisfaction. In fact, the literature review shows that there have been no such studies to date.

### 1.1. Self-Esteem and Life Satisfaction

Research conducted so far shows that self-esteem has a considerable impact on people’s psychological well-being [23] in all cultures examined [24]. For example, Moksnes and Espnes [14] found that self-esteem is not indifferent to how young people deal with challenges and take advantage of opportunities characteristic of their age. More precisely, when adolescents feel self-respect and believe themselves to be worthy, they also tend to report more effective functioning [25]. Rey et al. [25] observed a high correlation (*r* = 0.59) between self-esteem and life satisfaction among Spanish high school students. In a similar study, Pérez-Fuentes et al. [26] noticed that self-esteem played the role of facilitator in young people’s life satisfaction. A higher level of self-esteem corresponded with higher life satisfaction (*r* = 0.43). Marcionetti and Rossier [27] showed that a positive attitude toward the self has more impact on adolescents’ life satisfaction than general self-efficacy and belief in personal competence. Thus, adolescents’ liking or disliking themselves elicits a more positive evaluation of their lives than their beliefs in their own abilities or task capabilities. Diener and Diener [28] indicated that the correlation between self-esteem and life satisfaction among college students from 31 countries was at the level of *r* = 0.47. On the basis of the abovementioned research examples, we assumed that:

**Hypothesis** **1** **(H1).** *Positive evaluation of the self positively correlates with life satisfaction.*

### 1.2. Self-Esteem and Peer Communication

Peer-based connectedness and communication are crucial during adolescence, and influence young people’s mental well-being [29] and the quality of their satisfaction [30]. Communication with peers is a process that allows adolescents to reflect on their identity [31] and make sense of life experience [32]. Adolescents consider communication with their peers as a problem-solving resource [33] and spend much more time with other adolescents than with their parents [34,35]. They need to participate in sharing their feelings, beliefs, and ideas [36], thus building intimacy within peer relations [37].

Self-esteem is considered to be one of the crucial factors that impacts social development and functioning [38]. Rosenberg et al. [39] speak of self-esteem as a source of social behavior. According to Laible and colleagues [40], adolescents who report higher levels of self-worth feelings also express more secure attachments with peers than their counterparts with lower self-esteem. Moreover, various empirical studies confirm that self-esteem is significantly associated with peer communication [41]. This may be due to the fact that adolescents with high self-esteem better assess their relationships with friends as reliable and trusted [42]. Consequently, this can lead to better interpersonal communication with peers. For example, research on communication demonstrated that high self-esteem corresponds with self-disclosure [43,44], willingness to communicate [45,46], and lower levels of social anxiety [47]. Taking into account the previous research outcomes, we hypothesized that:

**Hypothesis** **2** **(H2).** *Self-esteem positively correlates with open peer communication, and negatively with difficulty in peer communication.*

### 1.3. Peer Communication and Life Satisfaction

Although during adolescence and emerging adulthood, young people expand their relationships beyond the family to peers and friendships, there has been scarce research addressing the links between peer communication and life satisfaction in this particular stage of life [48]. However, some studies on adolescents’ life satisfaction showed that variables related to the quality of interpersonal relationships are its strongest predictors [49,50]. Moreover, Piko and Hamvai [51] listed support from peers among the more important social correlates of adolescents’ life satisfaction. According to Tomé et al. [52], when young people maintain positive relationships with peers, using open communication, they also report higher psychological well-being and healthy behaviors. There is also some evidence that positive communication with peers may work as a protective factor against the increase of ill-being [53] and interpersonal distress [54]. Positive communication with peers significantly correlates with satisfaction among American adolescents [55]. Considering the above-mentioned theoretical and empirical premises, we supposed that:

**Hypothesis** **3** **(H3).** *Open peer communication positively correlates with life satisfaction, and difficult peer communication negatively correlates with life satisfaction.*

### 1.4. Peer Communication as a Mediator

The direct relationship between self-esteem and life satisfaction is well documented in the psychological literature. However, the mechanisms underlying this association are less known. To reduce this gap, we decided to verify whether communication with peers would serve as a mediator between both constructs.

A conceptual rationale behind choosing communication with peers as a potential mediator lies in the fact that the presence of appropriate and strong social skills may lead to positive outcomes, such as well-being or life satisfaction [56]. Moreover, communication with peers is considered one of the basic social abilities [57] and as such may affect mental health outcomes [58], decrease depression and anxiety [58], increase happiness [59] and life satisfaction [56], and contribute to the individual’s effectiveness [60].

Since life satisfaction depends on some crucial components as self-perception and interpersonal relationships [17], it is reasonable to assume that adolescents’ confident thoughts and feelings about their worth may have a higher effect on the positive evaluation of their lives as a whole when they openly communicate with their peers. In fact, open communication with peers was found to be pertinent to the development of a clear and coherent sense of the self among adolescents [61]. Conversely, the impact of an adequate self-esteem on the level of life satisfaction may be lower when the style of communication with peers is marked by imposing one’s opinion, making decisions for the other person, or exercising control over others. Another confirmation that communication may act as a mediator between self-esteem and life satisfaction comes from Kwan and colleagues’ research [62], which showed that maintaining harmony in interpersonal relationships played a mediatory role in the association between self-construals and life satisfaction. In other words, the individuals’ assessment of their value may impact the assessment of their relationship with others and together may contribute to satisfaction in life. Moreover, the choice of such a mediation model (self-esteem → peer communication → life satisfaction) is also justified by the fact that Poland occupies an intermediate position on the collectivism–individualism dimension [63,64]. Therefore, we expected self-esteem, communication with others, and life satisfaction to be not only mutually associated, but also in a mediation relationship. Following this premise, we hypothesized that:

**Hypothesis** **4** **(H4).** *Different styles of peer communication act as mediators between adolescents’ self-esteem and their life satisfaction.*

## 2. Materials and Methods

### 2.1. Participants and Procedure

The research was conducted among high school students in one of the Polish provincial capitals (*N* = 429). Around 59% of the participants were girls and 41% boys. The mean age was *M* = 17.45 (*SD* = 0.59) and the age range was between 16 and 18. It is characteristic of this research group that the students who participated in the study represented the late stage of adolescents. What distinguishes this developmental period is greater stability of self-esteem and increased communication with peers [40].

Data acquisition was based on the paper-and-pencil method. Informed and written consent of parents, legal guardians, and adolescents was obtained before starting the study. The adolescents were informed about the purpose of the study, its voluntary and confidential nature, and that they could stop participating at any time.

### 2.2. Rosenberg Self-Esteem Scale

The Rosenberg Self-Esteem Scale (RSES) is one of the most comprehensively used tools to evaluate global self-esteem perceived as an overall estimation of worthiness [65,66]. The RSES is a short, ten-item measure which contains five positively (e.g., “I take a positive attitude toward myself”) and five negatively (e.g., “All in all, I am inclined to feel that I am a failure”) worded statements. The negative items are reverse scored. The respondents are asked to denote on a scale from 1 to 4 how strongly they agree (1) or disagree (4) with each assertion. Similarly to other versions, the Polish adaptation by Łaguna et al. [67] has good psychometric properties and presents a good value of Cronbach’s alpha equivalent to 0.81 for the group of young people aged 14 to 18. In the current study, the internal consistency was excellent, showing α = 0.88.

### 2.3. Satisfaction with Life Scale

The Satisfaction with Life Scale (SWLS) authored by Diener et al. [68] and in the Polish adaptation by Juczyński [69] is one of the most often used self-report scales to measure individuals’ assessment of the quality of their lives based on their own unique criteria [70]. The one-dimensional scale includes five items (e.g., “In most ways my life is close to my ideal”) which are answered on a 7-point Likert scale (1 = strongly disagree and 7 = strongly agree). The scale range is between 5, meaning “extremely dissatisfied”, and 35, denoting “extremely satisfied”. SWLS is known for its good internal consistency. In the present study, the value of Cronbach’s alpha was 0.80.

### 2.4. Scale of Communication of Adolescents with Peers

The Scale of Communication of Adolescents with Peers (SCAP), developed by Napora [71], assesses the openness and difficulty in communication of adolescents with their peers. The subscale of openness relates to adolescents’ self-disclosure, spontaneous expression of feelings, thoughts, and beliefs (e.g., “It is not difficult for me to discuss my problems”). The subscale of difficulty in communication refers to adolescents’ domination, manifested by imposing one’s opinion and rules, deciding for another person what and how to do things, directing and having the last word in matters that concern both persons involved in the communication (e.g., “There are topics that I avoid in discussions”). The SCAP is a 20-item scale with 10 items for each factor. All of the items are answered on a 5-point Likert scale (1 = strongly disagree and 5 = strongly agree). The reliability and relevance of the tool are high. In Napora’s study, the value of Cronbach’s alpha was 0.90 for both the openness and difficulty subscales. In the current study, the value of Cronbach’s alpha was 0.85 for openness and 0.62 for difficulty.

### 2.5. Statistical Analysis

All descriptive, correlational, and regression statistical analyses were calculated with IBM SPSS Statistics version 20(IBM, Armonk, NY, USA). The variables included in the study were assessed for the normality of distribution. Based on the values of skewness and kurtosis lower than ±2 [72], Pearson’s correlation was applied. The variance inflation factor (VIF) was used to detect the degree of collinearity for all factors. A threshold value higher than 5.0 was assumed, in the present study, as an indicator of multicollinearity. Moreover, a tolerance value of 0.10 was implied as the suggested multicollinearity cut-off [73]. The Mahalanobis and Cook’s distance were implemented to verify the presence of outliers. The Mahalanobis distance was tested using Chi-square (χ^2^) and *p* < 0.001 as the criterion for the existence of outliers in the sample. With respect to Cook’s distance, case values should not exceed 1 [74].

Stepwise regression procedure was used to check for potential confounders. Only sex was included in Step 1, while self-esteem and both dimensions of communication were added in Step 2. The rationale for considering only sex as a theoretically and empirically relevant confounding variable is that several studies reported differences in self-esteem, life satisfaction, and communication with peers between women and men. For example, Birndorf et al. [75] showed, in a nationally representative longitudinal study, that fewer female adolescents than male adolescents reported high self-esteem. Similarly, Bachman et al. [76] found that in a large sample of 31,730 high school students, self-esteem scores were lower for girls than for boys. With respect to life satisfaction, several studies provided evidence that girls display a significant decrease in the subjectively perceived overall quality of life than boys [14]. In turn, there are also some intriguing findings that show females reporting higher satisfaction than boys [55]. The studies about communication between men and women are inconsistent [77]. On the one hand, females tend to report stronger peer attachment than males do [78], which can translate into better communication with others. Moreover, different socialization models can elicit different behavioral demands for both girls and boys [79]. While females’ conversations are often related to high-affiliation strategies (e.g., sharing information, support), males’ conversations consist of low-affiliation strategies (e.g., controlling, withdrawing) [80]. Due to the fact that the sample was largely homogeneous in terms of age, age was not taken into account as a possible confounder.

The mediation models (self-esteem → openness in communication → life satisfaction; self-esteem → difficulty in communication → life satisfaction) with 95% confidential intervals (CI) based on a 5000 bootstrap resampling were examined using Hayes PROCESS macro 3.4. (Heinrich-Heine-Universität, Düsseldorf, Germany) (Model 4) [81].

## 3. Results

### 3.1. Descriptive Statistics

Table 1 provides a summary of the arithmetic average (*M*), positive square root of the variance (*SD*), degree of skewness and kurtosis of self-esteem, life satisfaction, openness, and difficulty in communication.

None of the variables exceeded the values of skewness and kurtosis ±2, suggesting that the data was very close to a normal distribution. Consequently, a Pearson correlation analysis was undertaken.

### 3.2. Correlations

The results of the Pearson analysis (Table 2) showed statistically significant (*p* < 0.001) correlations between self-esteem, life satisfaction, openness in communication, and difficulty in communication. In line with the hypotheses, self-esteem correlated positively with life satisfaction (H1) and openness (H2), and negatively with difficulty in communication with peers (H2). Moreover, open peer communication was positively associated with life satisfaction, and difficult peer communication was negatively correlated with life satisfaction (H3). The observed magnitude of the correlation coefficients for the behavioral sciences [82] was between small and large.

In accordance with the presented outcomes, it can be assumed that higher levels of adolescents’ appraisal of their value coexists with higher subjective appraisal of their overall quality of life and open communication with peers. Additionally, difficulty in peer communication coincides with lower self-esteem and life satisfaction.

### 3.3. Multicollinearity and Confounding Variables

The multiple regression showed a VIF of 1.02–1.39 and a tolerance rate from 0.717 to 0.979. Both outcomes signify that there was no indication of multicollinearity for the variables. The Mahalanobis distance measure showed that there were no outliers in the set of the data since the lowest value of *p* was equal to 0.003643, being higher than *p* = 0.001. Moreover, Cook’s value between 0.000 and 0.052 was much lower than the cut-off of 1, thus confirming the lack of problematic cases. The process of accounting for covariates showed that sex did not make a considerable unique contribution to the model, explaining only 0.0231% of the variance (*R*^2^ = 0.000231) with *β* = 0.015, *t* = 0.314, and *p* = 0.753. The predictors of self-esteem and both dimensions of peer communication explained an additional 43% of the variance even after adjusting for the effect of sex.

### 3.4. Mediation Analyses

As regards H4, the two simple mediation analyses tested (1st model: self-esteem → openness in communication → life satisfaction; 2nd model: self-esteem → difficulty in communication → life satisfaction) indicated a significant effect.

**Figure 1:**
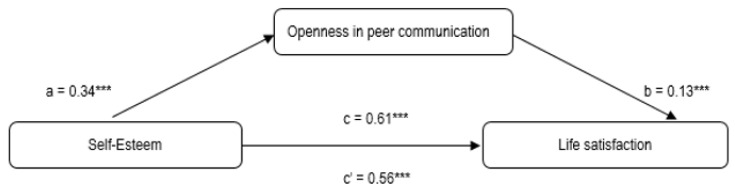
Results of mediation analysis of openness in peer communication in the relationship between self-esteem and life satisfaction; *** *p* < 0.001.

In the first model (Figure 1), statistically significant (*p* < 0.001) values of the regression coefficients were observed between self-esteem and openness in peer communication—path a (*β* = 0.34), and between openness in communication and life satisfaction—path b (*β* = 0.13). After including openness in peer communication as the mediator, the original value of the regression coefficient decreased from *β* = 0.61 (c) to *β* = 0.56 (c’), occupying the same significance level. The total indirect effect of self-esteem on life satisfaction was B(SE) = 0.0440 (0.0137) with 95%CI (0.0189; 0.0723), confirming that the association between self-esteem and life satisfaction was mediated by openness in peer communication.

**Figure 2:**
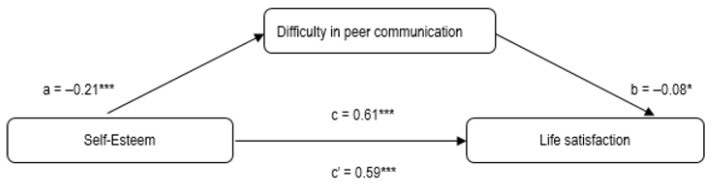
Results of mediation of difficulty in peer communication in the relationship between self-esteem and life satisfaction; *** *p* < 0.001; * *p* < 0.05.

In the second model (Figure 2), path a (*β* = −0.21) between self-esteem and difficulty in peer communication, and path b (*β* = −0.08) between difficulty in communication and life satisfaction, were also statistically significant. After considering difficulty in peer communication as the mediator, the value *β* = 0.61 (c) dropped slightly to *β* = 0.59 (c’), still remaining at the same significance level. The total indirect effect of self-esteem on satisfaction with life was B(SE) = 0.0166 (0.0091) with 95%CI (0.0005; 0.0358), corroborating the existence of a relationship between self-esteem and satisfaction with life mediated by difficulty in peer communication.

## 4. Discussion

In the present study, four hypotheses were verified and each of them found its support—evaluation of the self positively correlated with life satisfaction (H1); self-esteem positively correlated with open peer communication, and negatively with difficulty in peer communication (H2); open peer communication was positively associated with life satisfaction, and difficult peer communication was negatively linked to life satisfaction (H3); and both styles of peer communication acted as mediators between adolescents’ self-esteem and their life satisfaction (H4).

Firstly, a positive relationship between self-esteem and life satisfaction among adolescents corresponded to the role that self-evaluation plays in their psychological well-being, thus confirming both the theoretical premises and empirical outcomes obtained in other studies. Considering various developmental perspectives, Goldsmith et al. [83] indicated that personality traits related to self-confidence may be mentioned, along with the motivational and emotional dimensions, among the most important factors related to well-being and life satisfaction. Likewise, Steiger et al. [84] (p. 325) observed that self-esteem is pertinent to both “personal and social life outcomes.” Other studies and applied practice showed that adolescents’ self-understanding and self-worth were found to facilitate their process of adaptation to a new developmental context [14,85,86]. It is understandable that more positive feelings about the self co-occur with cognitive evaluation of one’s own life. An overall assessment of life reflects being confident and having a sense of self-worth because people who have high self-esteem respect themselves, do not consider themselves worse than others, acknowledge their limitations, and hope to mature and make progress in different dimensions of life [39].

Secondly, the positive correlation between self-esteem and open peer communication, and the negative correlation with difficult peer communication find their confirmation in previous research. For example, Richmond et al. [87] found that individuals’ evaluation of themselves was positively associated with their self-perceived communication ability in four categories of communication conditions (public speaking, talking in meetings, conversing in small groups, and talking to another person) with both known (e.g., acquaintances and friends) and unknown (e.g., strangers) people. In the same study, self-esteem was found to be one of the strongest predictors of self-perceived communication competence. In other analyses, McCroskey et al. [88] observed that low self-esteem negatively correlated with high levels of oral communication understanding across age and occupational groups. A more recent study [89] showed that young adults with high self-esteem and high agreeableness tend to self-disclose when meeting face-to-face and in online settings. Moreover, individuals who present higher levels of self-esteem tend to find communication more fulfilling and gratifying than their lower-self-esteem counterparts [90]. Taken together, the results of the present study support the sociometer and self-broadcasting perspectives that consider the effect of self-esteem on social relationships. Since people’s positive self-evaluations have social benefits [91] and are not indifferent to interpersonal relationships [92], it is understandable that adolescents’ feelings about themselves are associated with their peer communication.

Thirdly, satisfaction positively correlated with open communication, and correlated negatively with difficult communication thus referring to previous research that confirms the positive impact of interpersonal relationships on satisfaction with life. For example, Kim et al. [93] observed that adolescents’ perceived relationships with peers had a strong effect on their satisfaction over a three-year period. Lau and Bradshaw [94] found that time spent with friends and their social support correlated with overall life satisfaction. Schwarz and colleagues [95] verified that peer acceptance was positively linked to adolescents’ life satisfaction independently of their cultural background. In another study, Oh et al. [96] pointed out that companionship support, understood as a shared bond and a sense of belonging, was the only predictor of life satisfaction. Although none of the above-mentioned studies directly indicates the role of communication, but they rather show the influences of interpersonal relations in general, it can be concluded that connecting with peers is a meaningful factor of life satisfaction among adolescents. In fact, Richmond and Roach [97] (p. 104) observed that “willingness to communicate is the one, overwhelming communication personality construct which permeates every facet of an individual’s life and contributes significantly to the social, educational, and organizational achievements of the individual.” Moreover, the results obtained in the present study are in line with the social support theory. When adolescents establish connections with others through communication, they build a network of social contacts around them and thus enhance their psychological well-being [98].

Fourthly, the outcomes obtained in the current study indicate that both styles of peer communication are mediators between adolescents’ self-esteem and their life satisfaction. More explicitly, it can be assumed that adolescents with a high level of self-esteem who have communicative skills of self-disclosure and spontaneous sharing with peers consider their lives more satisfied. On the other hand, adolescents with lower self-esteem who have difficulty in communication with peers tend to assess their lives as less fulfilled. These findings confirm previous research, because in the psychological literature, satisfaction with life is considered an outcome of different psychological traits [21] and the social environment of friends [99,100]. Adolescents who positively evaluate themselves are confident and willing to start and maintain relationships with peers, which results in their better overall well-being, while adolescents who do not believe in themselves tend to withdraw from social interactions which, in turn, may lead to a lower overall satisfaction with life. The results obtained can be viewed from the perspective of the broaden-and-build theory [101] which suggests that a positive approach in the form of an emotion or a mindset increases people’s repertoires and shapes their personal resources. If high self-esteem is considered a psychological source of positive outcomes [102], it is very likely that it may lead to better communication and greater life satisfaction.

The most important implication of the present research is that it empirically supports the social cognitive theory, showing the importance of the interaction between personal characteristics and social skills for the general well-being of adolescents. The sense of personal adequacy, expressed in greater openness to interpersonal communication with others, may build a sense of satisfaction with life among young people on the threshold of emerging adulthood.

The practical connotation of this study relates to educational and/or therapeutic applications. Accompanying adolescents in the process of shaping and strengthening their own self-esteem in this stage of development may help them use their overall sense of self-worth in communicating with their peers and thus contribute to satisfaction in various dimensions of their functioning. The findings of the study go beyond the well-documented research that has focused on peer support, loneliness, and depressive symptomology. They show that the coexistence of components related to the self and others is important to adolescents’ quality of life.

## 5. Limitations

Several limitations of the current study should be listed. The first aspect we would like to highlight is the inclusion of a small number of sociodemographic variables that might serve as potential confounders. Future research could encompass variables such as the number of friends, time spent in face-to-face and on-line communication, sense of loneliness, and collectivism–individualism dimensions which seem to be important for life satisfaction. All of the above-mentioned variables could be tested for their possible impact on the direct relationship between self-esteem and life satisfaction among adolescents. Moreover, the study relied on a high school student sample of one of the Polish provincial capitals. In this sense, the obtained results cannot be generalized as they reflect only a small part of the whole group of adolescents. The most important limitation of this study is its cross-sectional design which does not allow us to draw causal conclusions on the direct and indirect relations between the studied variables. Therefore, the outcomes should be interpreted cautiously and, in the future, longitudinal or experimental research should be used to verify the obtained results.

## 6. Conclusions

The mediatory effect of peer communication suggests that the simple bivariate relationship between adolescents’ evaluation of the self and a subjective assessment of their overall quality of life may be more complex. It seems that self-esteem and life satisfaction, within this developmental group, are related because there are other phenomena which are not indifferent to the process of perceiving oneself and one’s life. An example of such a mechanism is the ability to establish relationships through open communication based on respect and honesty.

## Figures and Tables

**Table 1:** Descriptive statistics for self-esteem, life satisfaction, openness, and difficulty in communication (*N* = 429).

Variables	*M*	*SD*	Skewness	Kurtosis
Self-esteem	27.34	6.42	−0.205	−0.507
Life satisfaction	19.37	6.09	0.012	−0.553
Openness	37.10	7.34	−0.414	−0.420
Difficulty	25.21	6.07	0.012	−0.259

**Table 2:** Pearson correlation coefficients between self-esteem, life satisfaction, and both dimensions of peer communication (*N* = 429).

Scales	Self-Esteem	Life Satisfaction	Openness	Difficulty
Self-esteem	1			
Life satisfaction	0.64 ***	1		
Openness	0.30 ***	0.33 ***	1	
Difficulty	−0.23 ***	−0.22 ***	−0.49 ***	1

*** *p* < 0.001.

## Data Availability

The datasets used during this study are available from the corresponding author.
